# Dominance of CTX-M-Type Extended-Spectrum *β*-Lactamase (ESBL)-Producing *Escherichia coli* Isolated from Patients with Community-Onset and Hospital-Onset Infection in China

**DOI:** 10.1371/journal.pone.0100707

**Published:** 2014-07-01

**Authors:** Shu Xia, Xin Fan, Zengguang Huang, Liang Xia, Meng Xiao, Rongchang Chen, Yingchun Xu, Chao Zhuo

**Affiliations:** 1 State Key Laboratory of Respiratory Disease, the First Affiliated Hospital of Guangzhou Medical University, Guangzhou, China; 2 Department of Clinical Laboratory, Peking Union Medical College Hospital, Beijing, China; 3 The First Affiliated Hospital of Chongqing Medical University, Chongqing, China; St. Petersburg Pasteur Institute, Russian Federation

## Abstract

**Objective:**

To investigate CTX-M genotypes among extended-spectrum *β*-lactamase-producing *Escherichia coli* (ESBL-EC) isolated from patients with community-onset and hospital-onset infections in China, their clonality and the distribution of CTX-M variants in different specimens of community-onset and hospital-onset infections.

**Methods:**

ESBL-EC isolates were collected from general hospitals from 2011 to 2012 in China. Broth microdilution method antimicrobial susceptibility testing of 16 antibiotics was performed. Clinical data from community-onset and hospital-onset infections due to ESBL-EC were analyzed. ESBL-encoding genes were amplified by PCR and sequenced, and multilocus sequence typing (MLST) was performed for a random selection of predominant CTX-M type strains identified.

**Results:**

A total of 1,168 ESBL-EC isolates were obtained from various clinical specimens, 41.7% of which were responsible for causing community-onset infections. The presence of urinary calculi was higher in community-onset infections, whereas malignancy, cardiovascular and cerebrovascular diseases, dementia, chronic renal disease, diabetes mellitus and surgical treatment were found to have higher proportions in hospital-onset infections. There was no significant difference in trauma between community-onset and hospital-onset infections. 96.2% of the isolates were detected to harbor *bla*_CTX-M_ genes. *bla*_CTX-M-1_ group and *bla*_CTX-M-9_ group were detected at 40.7% and 48.7% respectively, and both positive group accounted for 10.6%. *bla*_CTX-M-55_ (24.8%) and *bla*_CTX-M-15_ (18.2%) were the major genotypes in *bla*_CTX-M-1_ group while *bla*_CTX-M-14_ (46.8%) was predominant in *bla*_CTX-M-9_ group. A comparison of *bla*_CTX-M_ distribution in different specimens between ESBL-EC causing community-onset and hospital-onset infection showed no significant difference. A total of 229 isolates were tested for MLST. ST131 (14%) was the predominant type. ST648, ST405 and ST1193 were also detected.

**Conclusions:**

Community-onset ESBL-EC has emerged as a common pathogen in China. CTX-M-14 is the most commonly encountered, CTX-M-55 and CTX-M-15 have spread rapidly. ST131 is the predominant clonal group, and the great diversity of CTX-M-producing isolates of *E. coli* has emerged in China.

## Introduction

The production of extended-spectrum *β*-lactamases (ESBLs) is one of the primary mechanisms conferring resistance to broad-spectrum *β*-lactam antibiotics. A large surveillance of antimicrobial resistance has shown that the detection rate of ESBLs in *Escherichia coli* has increased significantly over that in *Klebsiella pneumoniae* and exceeded 70% in mainland China [1]. A genotypic epidemiological study on ESBLs indicated that *bla*_CTX-M_ type genes have replaced *bla*_SHV_ and *bla*_TEM_ as the most common ESBL gene [2]. In addition, the prevalence of ESBL producing Enterobacteriaceae is increasing in community settings globally [3]. According to a recent study in the US, 36.8% of ESBL-producing *E. coli* (ESBL-EC) isolates were responsible for community-onset bloodstream infections (BSI) and urinary tract infections (UTI) [4]. Results from the Study for Monitoring Antimicrobial Resistance Trends (SMART) surveillance in European countries revealed that the prevalence of ESBL-EC causing community-onset intra-abdominal infections rose from 4% in 2002 to 7.4% in 2007 [5]. Surprisingly, a high incidence of ESBL-EC was also found in developing countries. A survey in rural Thai communities showed that faecal carriage of CTX-M-type ESBL-producing Enterobacteriaceae among asymptomatic individuals was up to 69.3% in 2010 [6]. The global spread of the *E. coli* ST131 clone was presumed to be partial responsible for the current situation [7].

Genotypic epidemiology data of ESBL-EC causing community-onset infections in China was insufficient. A multicenter study of the Ministry of Health National Antimicrobial Resistance Surveillance Net (MOHNARIN) in China during January 2007 to March 2008 revealed that 42.8% of urinary tract infections caused by ESBL-EC were community-onset infections [8]. However, there were few studies that compared the genotypic epidemiology of ESBL-EC isolated from community- and hospital-onset infections.

Previous regional surveys showed that CTX-M-14 and CTX-M-15 were the most common ESBLs produced by Enterobacteriaceae in China [2], [9], and some epidemic plasmids played a major role in the dissemination of *bla*_CTX-M-14_ and *bla*_CTX-M-15_ among *E. coli* and *K. pneumoniae* isolates [10], [11]. Nevertheless, the current genotypic epidemiology of ESBL-EC in China remains largely unknown. This study was proceeded to provide information on genotypic profile of CTX-M-type-carrying ESBL-EC in community- and hospital-onset infections and their clonality in mainland China.

## Materials and Methods

### Ethics Statement

This study protocol was approved by the Ethics Committee of The First Affiliated Hospital of Guangzhou Medical University. All subjects signed written informed consent prior to the study. Patient information was anonymized and de-identified prior to analysis.

### Bacterial Isolates and Data Collection

This was a national laboratory-based multicenter study of ESBL-EC genotypic epidemiology in 30 general hospitals of 24 provinces in mainland China. The hospitals were distributed in five regions across China: four in the northeast (Heilongjiang, Jilin, Liaoning and Inner Mongolia); five in the north (Beijing, Tianjin, Hebei, Shandong and Shanxi); five in the mid-east (Henan, Hubei, Zhejiang, Jiangxi and Anhui); five in the south (Sichuan, Yunnan, Guangxi, Guangdong and Hainan); and five in the northwest (Xinjiang, Gansu, Ningxia, Qinghai and Shaanxi). From July 2011 to July 2012, the first 60 non-repetitive ESBL-EC isolates (or all non-repetitive ESBL-EC isolates if the total number of non-repetitive ESBL-EC was less than 60) were sent to the Department of Clinical Laboratory in Peking Union Medical College Hospital, where they were stored at −80°C until further study. These bacterial isolates were isolated from different specimen types, including urine, blood, secretions, pus, bile, abdominal drainage fluid, bronchoalveolar lavage fluid and others.

Demographic and clinical information of the patients from whom ESBL-EC were isolated was retrieved retrospectively, which included age, gender, history of underlying diseases, specimen type, the date of patient admission and sample collection and so on. Community-onset infection was defined as an infection diagnosed within the first 48 h of hospitalization or outpatient service and no history of hospitalization within 30 days [12]. Hospital-onset infection was defined as an infection occurring more than 48 h after admission to the hospital. Health care associated infection as defined by Friedman [13] was included under hospital-onset infection.

### Antimicrobial Susceptibility Testing

ESBL-EC isolates were initially identified by routine methods in each participating hospital according to local operation procedures. When these “presumptive” ESBL-EC isolates were sent to the Department of Clinical Laboratory in Peking Union Medical College Hospital, production of ESBL was confirmed by broth microdilution method per Clinical and Laboratory Standards Institute (CLSI) guidelines [14]. The minimum inhibitory concentrations (MICs) of the following 16 drugs were determined with the broth microdilution method according to the CLSI performance standards [14]; imipenem (IPM), meropenem (MEM), ertapenem (EPM), tigecycline (TGC), colistin (CST), cefoperazone/sulbactam (CPS), piperacillin/tazobactam (TZP), cefepime (FEP), cefotaxime (CTX), ceftazidime (CAZ), ceftriaxone (CRO), aztreonam (ATM), amikacin (AMK), levofloxacin (LVX), ciprofloxacin (CIP), and minocycline (MIN). The results were interpreted by using the criteria of the CLSI for broth dilution [14]. *E. coli* ATCC 25922 was run in parallel for quality control, The interpretation breakpoint of cefoperazone/sulbactam (Pfizer Inc., USA) corresponded to that of cefoperazone, and that of tigecycline was based on the criteria proposed by the Food and Drug Administration (susceptibility at ≤2 mg/L).

### Molecular Detection of ESBL

All the isolates were screened by PCR with specific primers listed in Table S1 in File S1 for *bla*_CTX-M_ detection and group assignment, as described previously [15]. Detection of *bla*_TEM_ and *bla*_SHV_ was performed on both *bla*_CTX-M_-positive and *bla*_CTX-M_-negative samples. Purified PCR products were directly sequenced from both ends or cloned in pMD18-T and then sequenced. The DNA sequences and deduced amino acid sequences were analyzed using the NCBI BLAST program (http://www.ncbi.nlm.nih.gov/), and the subtypes of *β*-lactamase genes were confirmed by referring to the Lahey system (www.lahey.org/studies/).

### Multilocus Sequence Typing

Multilocus sequence typing (MLST) was performed for a random selection of predominant CTX-M type (including CTX-M-15, CTX-M-55 and CTX-M-14) strains identified in this study. MLST was performed with the seven conserved housekeeping genes (including *adk*, *fumC*, *gyrB*, *icd*, *mdh*, *purA* and *recA*). A detailed protocol of the MLST procedure, including allelic type and sequence type (ST) assignment methods, available at the University College Cork MLST website (http://mlst.ucc.ie/mlst/dbs/Ecoli), was used in this study.

### Statistical Analysis

The data were analyzed using SPSS 13.0 software package (SPSS, Chicago IL, USA). Continuous variables were compared using the Mann-Whitney U test. Qualitative variables were compared using the chi-square test or the Fisher’s exact test. *P*<0.05 was considered statistically significant.

## Results

### Distribution of ESBL-producing *E. coli*

In this study, 1,168 non-repetitive ESBL-producing *E. coli* isolates were obtained from clinical specimens. This included 221 isolates from the northeast (18.9%), 240 from the north (20.5%), 222 from the middle region (19.0%), 211 from the south (18.1%), and 274 from the northwest (23.5%). With regard to the source of the clinical specimens, the isolates were obtained from urine (47.9%), blood (22.5%), secretions (7.0%), pus (6.2%), bile (3.4%), abdominal drainage fluid (6.8%), bronchoalveolar lavage fluid (2.9%) and others (3.3%). Distribution of the isolates in community-onset and hospital-onset infection was listed in Table 1.

**Table 1:** Distribution of the isolates in community-onset and hospital-onset infection caused by Extended-Spectrum β-Lactamase–producing clinical *Escherichia coli* isolates (n = 1,168).

Specimen type	Community-onset infectionn(%)	Hospital-onset infectionn(%)
Urine	241(49.5)	319(46.8)
Blood	79(16.2)	184(27.0)
Secretions	34(7.0)	48(7.1)
Pus	48(9.8)	24(3.5)
Bile	19(3.9)	21(3.1)
Abdominal drainage fluid	42(8.6)	37(5.5)
Bronchoalveolar lavage fluid	13(2.7)	20(2.9)
Others	11(2.3)	28(4.1)
Total	487(100.0)	681(100.0)

A comparison of clinical data in community-onset versus hospital-onset infection groups was shown in Table 2. In the two groups, 78.2% and 38.5% of patients had no history of underlying diseases when admitted to hospital. The presence of urinary calculi was higher in community-onset infections, whereas common underlying diseases including malignancy, cardiovascular and cerebrovascular diseases, dementia, chronic renal disease, diabetes mellitus and surgical treatment were found to have higher proportions in hospital-onset infections. There was no statistically significant difference in trauma between community-onset infections and hospital-onset infections.

**Table 2:** Characteristics of community-onset and hospital-onset patients infected with extended-spectrum β-lactamase-producing *Escherichia coli.*

Characteristics	Community-onset (n = 487) % (n)	Hospital-onset (n = 681) % (n)	*P*Value
Male	47.4(231)	43.2(294)	0.111
Age (year)	52.3±19.3	58.6±18.4	<0.001
**No history of underlying diseases**	78.2(381)	38.5(262)	<0.001
**Underlying diseases**			
Malignancy	2.311)	18.9(129)	<0.001
Chronic renal disease	3.7(18)	10.0(68)	<0.001
Urinary tract calculi	5.8(28)	1.8(12)	<0.001
Cardiovascula and cerebrovascular diseases	1.0(5)	10.7(73)	<0.001
Dementia	0.2(1)	5.1(35)	<0.001
Diabetes mellitus	2.3(11)	5.1(35)	0.013
Trauma	5.3 (26)	5.6(38)	0.858
Surgical treatment	1.4(7)	9.4(64)	<0.001

### Antimicrobial Susceptibility Testing

All the ESBL-EC isolates had high susceptibility rates to imipenem (97.5%), meropenem (99.2%), ertapenem (95.1%), tigecycline (99.0%), colistin (95.1%), piperacillin/tazobactam (89.3%) and amikacin (88.4%). Regarding MIC value, the lowest MIC_90_ values were seen in meropenem (≤0.06 mg/L), imipenem (0.5 mg/L), ertapenem (0.5 mg/L), tigecycline (0.5 mg/L) and colistin (1 mg/L), followed by piperacillin/tazobactam (32 mg/L) and amikacin (16 mg/L). A majority of of ESBL-EC isolates were resistant to ceftazidime (58.6%), cefepime (91.7%), aztreonam (84.4%) and ciprofloxacin (81.2%). All the isolates were resistant to cefotaxime and ceftriaxone.

In comparison of the drug susceptibility of ESBL-EC from hospital- and community-onset infections, it revealed that there were similar resistant profiles, and no statistically significant difference was found for any drug (P>0.05) (see Table S2 in File S1).

### Molecular Detection of ESBL

96.2% (1,124/1,168) of the isolates were detected to harbor *bla*_CTX-M_ genes, and *bla*_CTX-M-1_ and *bla*_CTX-M-9_ positive groups were detected at 40.7% (457/1,124) and 48.7% (547/1,124), respectively, and the *bla*_CTX-M-1_ and *bla*_CTX-M-9_ double-positive group accounted for 10.6% (120/1,124). *bla*_CTX-M-2_, *bla*_CTX-M-8_, and *bla*_CTX-M-25_ groups were not detected in the isolates. In the *bla*_CTX-M-1_ group, *bla*_CTX-M-55_ and *bla*_CTX-M-15_ were the major genotypes, accounting for 48.4% (221/457) and 35.7% (163/457), respectively; the remaining genotypes included *bla*_CTX-M-3_, *bla*_CTX-M-64_, *bla*_CTX-M-123_, *bla*_CTX-M-101_ and *bla*_CTX-M-132_. In the *bla*_CTX-M-9_ group, *bla*_CTX-M-14_ was the major genotype, accounting for 79.7% (436/547); the remaining included *bla*_CTX-M-27_, *bla*_CTX-M-65_, *bla*_CTX-M-24_, *bla*_CTX-M-125_, and *bla*_CTX-M-104_.

In the *bla*_CTX-M_-positive isolates, TEM-type ESBLs were not detected. TEM PCR amplification was positive in 1011 strains, in which 998 isolates were confirmed to be TEM-1, 8 TEM-105, 5 TEM-like by sequence analysis. Ten strains harbored an SHV-type ESBLs in the *bla*_CTX-M_-positive isolates: SHV-12 (n = 7) and SHV-5 (n = 3). Among 44 *bla*_CTX-M_-negative isolates, 11 of them were detected to harbor *bla*_TEM-1_ (n = 11), and also produce *bla*_SHV-12_ (n = 9) and *bla*_SHV-5_ (n = 2). *bla*_TEM_ and *bla*_SHV_ type ESBLs were not detected in the remaining 33 isolates.

A comparison of *bla*_CTX-M_ distribution in different specimens between ESBL-EC causing community-onset and hospital-onset infections showed that no significant difference was noted with regard to the proportions of different types of ESBLs between the two groups (Table 3).

**Table 3:** CTX-M-type genes distribution of ESBL-EC in various types of Specimen across China.

	Community-onset infection n(%)								Hospital-onset infection n(%)							
Specimentype	Urine	Blood	Secretions	Pus	Bile	ADF	BALF	Others	Urine	Blood	Secretions	Pus	Bile	ADF	BALF	Others
**CTX-M-1** **group**	**107**	**27**	**15**	**14**	**5**	**19**	**4**	**5**	**121**	**68**	**23**	**9**	**8**	**15**	**4**	**13**
CTX-M-55	52(48.6)	10(37.0)	6(40.0)	8(57.1)	2(40.0)	6(31.6)	0(0.0)	2(40.0)	63(52.0)	35(51.4)	13(56.5)	6(66.7)	3(37.5)	7(46.7)	2(50.0)	6(46.1)
CTX-M-15	37(34.6)	11(40.8)	3(20.0)	6(42.9)	3(60.0)	10(52.6)	1(25.0)	0(0.0)	44(36.4)	21(30.9)	9(39.1)	2(22.2)	5(62.5)	5(33.3)	1(25.0)	5(38.5)
CTX-M-3	12(11.2)	4(14.8)	1(6.7)	0(0.0)	0(0.0)	2(10.5)	0(0.0)	2(40.0)	8(6.6)	8(11.8)	0(0.0)	0(0.0)	0(0.0)	2(13.3)	1(25.0)	1(7.7)
Others	6(5.6)	2(7.4)	5(33.3)	0(0.0)	0(0.0)	1(5.3)	3(75.0)	1(20.0)	6(5.0)	4(5.9)	1(4.4)	1(11.1)	0(0.0)	1(6.7)	0(0.0)	1(7.7)
**CTX-M-9 group**	**103**	**31**	**15**	**31**	**7**	**18**	**5**	**4**	**164**	**91**	**19**	**12**	**10**	**16**	**11**	**10**
CTX-M-14	82(79.7)	26(84.0)	12(80.0)	25(80.6)	6(85.7)	16(89.0)	4(80.0)	1(25.0)	125(76.2)	75(82.4)	15(79.0)	10(83.4)	7(70.0)	13(81.3)	9(81.8)	10(100.0)
CTX-M-27	9(8.7)	2(6.4)	1(6.7)	1(3.2)	1(14.3)	1(5.5)	1(20.0)	1(25.0)	17(10.4)	4(4.4)	2(10.5)	1(8.3)	3(30.0)	0(0.0)	1(9.1)	0(0.0)
CTX-M-65	10(9.7)	2(6.4)	2(13.3)	2(6.5)	0(0.0)	1(5.5)	0(0.0)	1(25.0)	13(7.9)	6(6.6)	2(10.5)	0(0.0)	0(0.0)	1(6.2)	1(9.1)	0(0.0)
Others	2(1.9)	1(3.2)	0(0.0)	3(9.7)	0(0.0)	0(0.0)	0(0.0)	1(25.0)	9(5.5)	6(6.6)	0(0.0)	1(8.3)	0(0.0)	2(12.5)	0(0.0)	0(0.0)
**CTX-M-1** **and** **CTX-M-9** **group**	**29**	**10**	**2**	**3**	**5**	**5**	**3**		**28**	**15**	**4**	**3**	**1**	**4**	**4**	**4**
CTX-M-55 andCTX-M-14	9(31.0)	4(40.0)	2(100.0)	2(66.7)	2(40.0)	1(20.0)	2(66.7)	0(0.0)	13(46.4)	4(26.7)	3(75.0)	2(66.7)	0(0.0)	1(25.0)	1(25.0)	3(75.0)
CTX-M-15 andCTX-M-14	8(27.6)	5(50.0)	0(0.0)	1(33.3)	1(20.0)	2(40.0)	0(0.0)	0(0.0)	7(25.0)	4(26.7)	1(25.0)	1(33.3)	1(100.0)	2(50.0)	1(25.0)	0(0.0)
Others	12(41.4)	1(10.0)	0(0.0)	0(0.0)	2(40.0)	2(40.0)	1(33.3)	0(0.0)	8(28.6)	7(46.6)	0(0.0)	0(0.0)	0(0.0)	1(25.0)	2(50.0)	1(25.0)

Abbreviations: ADF: abdominal drainage fluid; BALF: bronchoalveolar lavage fluid.

The five study regions showed certain differences in the *bla*_CTX-M_ distribution of ESBL-EC. The proportion of *bla*_CTX-M-1_ group in south China was less than 32%, which was significantly lower than those in the other four regions (*P*<0.01). The proportion of *bla*_CTX-M-55_ in *bla*_CTX-M-1_ group was lower in northwest and south China (41.0%) than in the northeast, north, and mid-east (50%), but the differences were not statistically significant (*P*>0.05). The proportion of *bla*_CTX-M-9_ group was the highest in south China (55.6%; it was approximately 45% in the other four regions), but the differences among the five regions were not statistically significant (*P*>0.05). The proportion of *bla*_CTX-M-14_ was similar in the five regions (*P*>0.05). The detailed results are shown in Table S3 in File S1.

### Multilocus Sequence Typing

A total of 229 isolates (of 34 CTX-M-15 strains, 86 CTX-M-55 strains and 109 CTX-M-14 strains) were tested. Generally, ST131 (14%, 32 isolates) was the predominant type, followed by ST648 (10.1%, 23 isolates), ST405 (8.3%, 19 isolates) and ST1193 (7.9%, 18 isolates). Other sequence types like ST38, ST3177, ST10, ST2003, ST95, ST410, ST393, ST354, ST155 and ST617 and so on were also detected in the present study. In the CTX-M-15 strains, clonal group ST405 (20.6%) was the predominant type, followed by ST167 (14.7%) and ST131 (11.8%), while in the CTX-M-55 strains, ST1193 (12.5%) was the predominant clonal group, followed by ST131 (8.2%). In the CTX-M-14 strains, clonal group ST131 (19.3%) was the predominant, and ST648 (18.3%) ranked second. 32 ST131 ESBL-EC strains were isolated from both community-onset infections (34.4%) and hospital-onset infections (65.6%), and they were mostly obtained from urine (50.0%) and blood (25.0%).

## Discussion

To our knowledge, this work represents the first national large-scale study on the clinical information, antimicrobial resistance and genotype epidemiology of ESBL-EC in China.

Greater than 50% ESBL-EC isolates in this study were resistant to all the third-generation cephalosporins, cefepime, and fluoroquinolones. It has been suggested that cephalosporin and fluoroquinolone are not considered effective choices for treatment of patients with ESBL-producing Enterobacteriaceae infection because of relatively high mortality associated with inappropriate therapy [16]. With regard to β-lactam/β-lactamase inhibitor combinations, ESBL-EC had low resistance rates to piperacillin/tazobactam and cefoperazone/sulbactam (<20%) in this study, which suggest they might be appropriate drugs against non–BSI caused by ESBL, though there is controversy surrounding whether β-lactam/β-lactamase–inhibitor combinations are a good option [16], [17]. As expected, the ESBL-EC had high susceptibility rates to imipenem, meropenem, and ertapenem (94.8–99%), which suggest that carbapenems are considered as last resort antibiotics for the treatment of ESBL-producing *E. coli* infections in China.

The prevalence of ESBL-EC isolates in community-onset infections has been increasing worldwide. Similar results were found in the present study. Of community-onset UTI, BSI and IAI infections, ESBL-EC took up a notable proportion (19.4%–33.7%), which was in accordance with those reported in Europe and America [3], [4], [12]. Many risk factors have been associated with the prevalence of ESBL-EC isolates in the community. For example, recurrent UTI and dependent functional status were associated with isolation of CTX-M *E. coli* responsible for community-onset UTI [3], which correlated with the results of this study, where 11.2% patients in the community-onset UTI group had a history of urinary tract calculus, a risk factor for and consequence of recurrent UTI [18]. Previous exposure to second or third-generation cephalosporins has been considered as an important risk factor for the occurrence of ESBL-producing Enterobacteriaceae in community-onset infection by many researchers [3], [19], [20]. Although the number and distribution of community health-care settings, e.g. nursing homes, in China are not comparable to developed countries, the frequency and proportion of prescribed antibiotics in the rural areas of China are higher compared with the developed countries, and the number of antibiotics per 100 prescriptions was 54.62, and personal use of broad-spectrum penicillins or cephalosporins and fluoroquinolones is common [21]. This potentially may have contributed to the high incidence of ESBL-EC in community-onset infections.

A 2010–2011 survey in the United States showed that CTX-M type accounted for 85.4% of ESBL-producing *E. coli* strains [3]. In China, CTX-M–type ESBLs accounted for more than 70% ESBL-producing *E. coli* strains over the past 10 years; CTX-M-14 remains the most abundant genotype, although the detection rate of CTX-M-15 has shown a continuously increasing trend in recent years [9]–[11]. Similar findings were obtained in the present study, in that the proportion of CTX-M–producing *E. coli* had increased to 96.2%, and CTX-M-14 remained the most common genotype of ESBLs, accounting for 46.8% of isolates. CTX-M-55, a variant of CTX-M-15 by one amino acid substitution of Ala-77-Val [22], was scarcely found in clinical isolates previously [22], [23]. However, the prevalence of CTX-M-55 (24.8%) has increased significantly and become the secondary genotype of ESBLs, following CTX-M-14 in this study. Interestingly, CTX-M-55 was first reported to be highly prevalent (25%) in ESBL-producing *E. coli* isolated from the intestinal samples of pets in China in 2010 [24]. Subsequent surveillance indicated that CTX-M-55 was predominant amongst ESBL-EC from animal sources [25]–[27]. In addition, CTX-M-27, CTX-M-65 and CTX-M-24 commonly identified from animal derived isolates accounted for a notable proportion in clinical isolates, as discovered in the present and from previous studies [25]–[27]. The distribution of the above ESBLs was generally the same between hospitals and communities and across different regions in China. The results may suggest that the injudicious use of antimicrobial agents amongst livestock and hospitals may result in mutation and subsequent epidemiological change of major ESBL genotypes circulated (e.g. CTX-M-3 to CTX-M-15 and CTX-M-55; CTX-M-14 to CTX-M-27 and CTX-M-65), and further lateral transfer of resistant genes between different isolates. Whether drug resistance genes are transmitted between animal- and human-derived *E. coli* via plasmids needs to be investigated. The lateral transfer of resistant genes between animals and humans may also contribute to the rising prevalence of ESBL-EC in community, and *E. coli* from healthy food animals can be important reservoirs of *bla*_CTX-M_ genes and may contribute to the dissemination and transfer of these *β*-lactamase genes throughout China.

In the present study, the *bla*_CTX-M-64_, *bla*_CTX-M-123_, and *bla*_CTX-M-132_ genes, new mutant genotypes containing at least three hybrid sites of CTX-M-14 and CTX-M-15 [28], were first detected in clinical isolates in China; the three genotypes were first found in animal isolates in China [29]. Although the detail mechanisms of resistance remain unknown, it is possible that this may be due transmission of isolates and their plasmids between animals and humans, and remains an area for future studies.

The majority of CTX-M–producing *E. coli* particularly CTX-M-15-producing *E. coli* belonged to a specific clone defined as MLST profile ST131 in many countries [30], [31], which was presumed to be some reason for the growing problem of antibiotic resistance. Same as previous research, ST131 (14%) was the predominant clonal group established among these strains. It was mostly detected in the CTX-M-14 strains, but also accounted for a significant proportion in CTX-M-15 and CTX-M-55 strains. What’s more, the majority of ST131 strains were obtained from urine (50.0%), which may be connected with its variable virulence potential [32]. In addition, the finding of non-ST131 profile clones like ST405, ST167, ST 3177, ST1193, and ST648 and so on showed the great diversity of CTX-M-producing isolates of *E. coli* had emerged in China.

There were some limitations in this study. Firstly, some of isolates were not susceptible to carbapenems, it would be important to know if some of these isolates produce carbapenemases. However, the related experiment is ongoing by one of our collaborative researching group and they plan to report our results in a separate paper. Secondly, some clinical information such as previous antibiotic treatment, medical exposure (e.g., received renal dialysis, nasogastric tube use), and outcome of the patients, were not acquired. The lack of this data made it impossible to analyze the risk factors for ESBL-EC infections in the same manner as preformed previously. However, the acquired data makes it possible to classify the all the cases into community-onset and hospital-onset infection, and the large sample size provide some clues of the risk factors for ESBL-EC infections in China. It would be worthwhile in future investigations to collect more detailed clinical data to learn in a more comprehensive manner the risk factors for different CTX-M variants in China. In addition, it will be valuable to discuss zoonotic transmission routes and possible transmission of *bla*_CTX-M_ positive *E. coli* between human and animals in China. It is regrettable that we could not obtain some data now. We hope to examine ESBL-EC isolated from both human and animals in parallel study in the follow-up research.

In summary, this study examined the drug resistance and genotypic epidemiology of 1,168 clinical isolates of ESBL-producing *E. coli*. We found that the CTX-M type is still the primary genotype of ESBL in China and that its proportion (96.2%) has increased. The specific genotype of CTX-M has undergone great changes, and it is worth noting that main resistant genotypes, represented by CTX-M-55 and CTX-M-15, have spread rapidly. ST131 (14%) was the predominant clonal group, and at the same time the great diversity of CTX-M-producing isolates of *E. coli* has emerged in China. Further studies should continue to monitor ESBL-producing bacteria and explore the mechanisms of spread of CTX-M genotypes in these bacteria to provide reference data to enable relevant infection control.

## Supporting Information

File S1 **Contains the files: Table S1.** Primers used for polymerase chain reaction amplification of bla genes in this study. Notes: Degenerate bases, K for G or T; R for A or G; S for C or G; Y for C or T. **Table S2.** Susceptibility of extended-spectrum β-lactamase-producing *Escherichia coli* (ESBL-EC) isolates against common antimicrobial agents and comparison of antimicrobial susceptibility of Extended-Spectrum β-Lactamase–producing Escherichia coli causing Community-onset and Hospital-onset infection. Abbreviations: Imipenem (IPM), Meropenem (MEM), Ertapenem (EPM), Tigecycline (TGC), Colistin (CST), Cefoperazone/sulbactam (CPS), Piperacillin/tazobactam (TZP), Cefepime (FEP), Cefotaxime (CTX), Ceftazidime (CAZ), Ceftriaxone (CRO), Aztreonam (ATM), Amikacin (AMK), Levofloxacin (LVX), Ciprofloxacin (CIP), and Minocycline (MIN). **Table S3.** The CTX-M genotypic distribution of extended-spectrum β-lactamases-producing *E. coli* in various regions across China.
(DOCX)
