# Supplementary material for: Dominance of CTX-M-Type Extended-Spectrum β-Lactamase (ESBL)-Producing Escherichia coli Isolated from Patients with Community-Onset and Hospital-Onset Infection in China
Source: PLoS One. 2014 Jul 1;9(7):e100707. doi: 10.1371/journal.pone.0100707 (PMC4077569; doi:10.1371/journal.pone.0100707)
Supplement: File S1 — Contains the files: Table S1. Primers used for polymerase chain reaction amplification of bla genes in this study. Notes: Degenerate bases, K for G or T; R for A or G; S for C or G; Y for C or T. Table S2. Susceptibility of extended-spectrum β-lactamase-producing Escherichia coli (ESBL-EC) isolates against common antimicrobial agents and comparison of antimicrobial susceptibility of Extended-Spectrum β-Lactamase–producing Escherichia coli causing Community-onset and Hospital-onset infection. Abbreviations: Imipenem (IPM), Meropenem (MEM), Ertapenem (EPM), Tigecycline (TGC), Colistin (CST), Cefoperazone/sulbactam (CPS), Piperacillin/tazobactam (TZP), Cefepime (FEP), Cefotaxime (CTX), Ceftazidime (CAZ), Ceftriaxone (CRO), Aztreonam (ATM), Amikacin (AMK), Levofloxacin (LVX), Ciprofloxacin (CIP), and Minocycline (MIN). Table S3. The CTX-M genotypic distribution of extended-spectrum β-lactamases-producing E. coli in various regions across China. (DOCX) [file pone.0100707.s001.docx]

**Supporting Information**

**Table S1** Primers used for polymerase chain reaction ampliﬁcation of bla genes in this study.

| Target group | DNA sequence (5'-3') | GenBank accession No. |
| --- | --- | --- |
| *bla*_CTX-M_ | F: ATGTGCAGYACCAGTAARGTKATGGC  R: TGGGTRAARTARGTSACCAGAAYCAGCGG | AY458016 |
| *bla*_CTX-M-1_ | F: CAGCGCTTTTGCCGTCTAAG  R: GGCCCATGGTTAAAAAATCACTGC | AY571969 |
| *bla*_CTX-M-2_ | F: CGACGCTACCCCTGCTATT  R: CCAGCGTCAGATTTTTCAGG | X92507 |
| *bla*_CTX-M-8_ | F: TCGCGTTAAGCGGATGATGC  R: AACCCACGATGTGGGTAGC | AF189721 |
| *bla*_CTX-M-9_ | F: GCGCATGGTGACAAAGAGAGTGCAA  R: GTTACAGCCCTTCGGCGATGATTC | AF174129 |
| *bla*_CTX-M-25_ | F: GCACGATGACATTCGGG  R: AACCCACGATGTGGGTAGC | AF518567 |
| TEM | F: GAGTATTCAACATTTTCGT  R: ACCAATGCTTAATCAGTGA | AY458016 |
| SHV | F: CGCCGGGTTATTCTTATTTGTCGC  R: TCTTTCCGATGCCGCCGCCAGTCA | X98101 |

Notes: Degenerate bases, K for G or T; R for A or G; S for C or G; Y for C or T.

**Table S2** Susceptibility of extended-spectrum β-lactamase-producing *Escherichia coli* (ESBL-EC) isolates against common antimicrobial agents and comparison of antimicrobial susceptibility of Extended-Spectrum β-Lactamase–producing Escherichia coli causing Community-onset and Hospital-onset infection.

| Agent | ESBL-EC isolates  (n=1,168) | |  | Community-onset infection (n=487) | |  | Hospital-onset infection (n=681) | |
| --- | --- | --- | --- | --- | --- | --- | --- | --- |
|  | Resistant  (%) | Susceptible  (%) |  | Resistant  (%) | Susceptible  (%) |  | Resistant  (%) | Susceptible  (%) |
| IPM | 1.0 | 97.5 |  | 1.0 | 97.3 |  | 1.0 | 97.7 |
| MEM | 0.4 | 99.2 |  | 0.4 | 99.4 |  | 0.4 | 99.1 |
| EPM | 2.4 | 95.1 |  | 2.7 | 95.9 |  | 2.2 | 94.6 |
| TGC | 0.4 | 99.0 |  | 0.6 | 98.8 |  | 0.3 | 99.1 |
| CST | 4.9 | 95.1 |  | 5.3 | 94.7 |  | 4.6 | 95.4 |
| CPS | 15.2 | 61.6 |  | 15.0 | 63.9 |  | 15.4 | 59.9 |
| TZP | 4.5 | 89.3 |  | 4.1 | 90.8 |  | 4.8 | 88.3 |
| FEP | 91.7 | 4.7 |  | 92.4 | 4.3 |  | 91.2 | 5.0 |
| CTX | 100.0 | 0.0 |  | 100.0 | 0.0 |  | 100.0 | 0.0 |
| CAZ | 58.6 | 33.2 |  | 58.7 | 32.0 |  | 58.4 | 34.1 |
| CRO | 100.0 | 0.0 |  | 100.0 | 0.0 |  | 100.0 | 0.0 |
| ATM | 84.4 | 15.3 |  | 86.0 | 14.0 |  | 83.3 | 16.3 |
| AMK | 8.6 | 88.4 |  | 7.4 | 89.7 |  | 9.4 | 87.4 |
| LVX | 74.3 | 19.2 |  | 72.9 | 20.1 |  | 75.3 | 18.5 |
| CIP | 81.2 | 16.6 |  | 79.9 | 18.1 |  | 82.1 | 15.6 |
| MIN | 33.2 | 54.6 |  | 33.9 | 54.2 |  | 32.7 | 54.9 |

Abbreviations: Imipenem (IPM), Meropenem (MEM), Ertapenem (EPM), Tigecycline (TGC), Colistin (CST), Cefoperazone / sulbactam (CPS), Piperacillin / tazobactam (TZP), Cefepime (FEP), Cefotaxime (CTX), Ceftazidime (CAZ), Ceftriaxone (CRO), Aztreonam (ATM), Amikacin (AMK), Levofloxacin (LVX), Ciprofloxacin (CIP), and Minocycline (MIN).

**Table S3** The CTX-M genotypic distribution of extended-spectrum β-lactamases-producing *E. coli* in various regions across China.

| Region | Northeast (n=221) | North  (n=240) | Mid-east  (n=222) | South  (n=211) | Northwest  (n=274) |
| --- | --- | --- | --- | --- | --- |
| **CTX-M type** | **218 (98.6)** | **233 (97.1)** | **216 (97.3)** | **188 (89.1)** | **269 (98.2)** |
| **CTX-M-1 group** | **91 (41.7)** | **95(40.8)** | **98 (45.4)** | **60 (31.9)** | **113 (42.0)** |
| CTX-M-55 | 45 (49.5) | 50 (52.6) | 55 (56.2) | 24(40.0) | 47 (41.6) |
| CTX-M-15 | 29 (31.9) | 35 (36.8) | 28 (28.6) | 24 (40.0) | 47 (41.6) |
| CTX-M-3 | 11 (12.1) | 6(6.3) | 6 (6.1) | 10 (16.6) | 8 (7.0) |
| CTX-M-64 | 4 (4.3) | 1 (1.1) | 6 (6.1) | 1 (1.9) | 3 (2.7) |
| CTX-M-123 | 2（2.2） | 3 (3.2) | 2 (2.0) | 0 (0.0) | 5 (4.4) |
| CTX-M-101 | 0 (0.0) | 0 (0.0) | 1 (1.0) | 0 (0.0) | 3 (2.7) |
| CTX-M-132 | 0 (0.0) | 0 (0.0) | 0 (0.0) | 1 (1.9) | 0 (0.0) |
| **CTX-M-9 group** | **106 (48.6）** | **109（46.8）** | **99 (45.8)** | **109 (58.0)** | **124 (46.1)** |
| CTX-M-14 | 87 (82.0） | 86（78.9） | 75 (75.8) | 88 (80.7) | 100 (80.6) |
| CTX-M-27 | 4 (3.8） | 7（6.4） | 12 (12.1) | 14 (12.9) | 8 (6.5) |
| CTX-M-65 | 11 (10.4） | 7（6.4） | 9 (9.1) | 5 (4.6) | 9 (7.3) |
| CTX-M-24 | 3 (2.8） | 6（5.6） | 0 (0.0) | 2 (1.8) | 2 (1.6) |
| CTX-M-125 | 1 (1.0） | 1 (0.9) | 1 (1.0) | 0 (0.0) | 1 (0.8) |
| CTX-M-104 | 0 (0.0) | 1 (0.9) | 1 (1.0) | 0 (0.0) | 3 (2.4) |
| CTX-M-13 | 0 (0.0) | 0 (0.0) | 0 (0.0) | 0 (0.0) | 1 (0.8) |
| CTX-M-17 | 0（0.0） | 0（0.0） | 1 (1.0) | 0（0.0） | 0（0.0） |
| CTX-M-157 | 0（0.0） | 1（0.9） | 0（0.0） | 0（0.0） | 0（0.0） |
| **CTX-M-1 and CTX-M-9 group** | **21（9.6）** | **29（12.4）** | **19（8.8）** | **19（9.0）** | **32（11.9）** |
| CTX-M-55 and CTX-M-14 | 8（38.0） | 12（41.4） | 9（47.4） | 8（42.1） | 12(37.5) |
| CTX-M-15 and CTX-M-14 | 5（24.0） | 9（31.0） | 5（26.3） | 6（31.6） | 9(28.1) |
| others | 8（38.0） | 8（27.6） | 5（26.3） | 5（26.3） | 11(31.4) |
